# Opioid-induced hyperalgesia: Are thalamic T-type calcium channels treatment targets?

**DOI:** 10.1172/JCI165977

**Published:** 2022-12-15

**Authors:** Slobodan M. Todorovic

**Affiliations:** 1 Department of Anesthesiology and; 2 Neuroscience and Pharmacology Graduate Program; University of Colorado, Anschutz Medical Campus, Aurora, Colorado, USA.

## Abstract

Opioid-induced hyperalgesia (OIH) is a state of paradoxically enhanced pain transmission, termed nociceptive sensitization, described to occur in both humans and animals after repeated administration of opioid drugs, including rapidly acting remifentanil. However, molecular mechanisms of OIH remain understudied. In this issue of the *JCI*, Yan Jin and colleagues provided strong evidence that hyperexcitable thalamocortical networks drive remifentanil-induced hyperalgesia in a rodent model of postsurgical pain. Furthermore, the authors specifically identified an important role of the Ca_V_3.1 isoform of low-voltage-activated or T-type calcium channels (T-channels) in this process. Further experiments are needed to determine whether thalamic T channels could serve as targets for the treatment of OIH.

## Opioid-induced hyperalgesia

The use of opioids in operating rooms and clinics has steadily increased over the last decades, making this drug class one of the most commonly prescribed in the USA. Most commonly used injectable general anesthetics do not provide the level of analgesia required for surgical procedures, necessitating the use of other agents, such as opioid analgesics, in the perioperative period. Specifically, use of opioids with fast pharmacokinetics, such remifentanil, are desirable in the operating rooms in conjunction with intravenous anesthetics, such as propofol, since they can be titrated to desired levels of analgesia and cardiovascular parameters. Furthermore, since remifentanil is rapidly eliminated from the blood plasma following completion of the infusion, its effects quickly dissipate, even after very long infusions. Although opioids are effective in treating the acute pain associated with surgical procedures, they are only partially effective for more chronic painful disorders, and their use is associated with side effects including constipation, urinary retention, impaired cognitive function, respiratory depression, tolerance, addiction, and OIH. OIH (1, 2) is particularly problematic because it sensitizes pain-sensing neurons (nociceptors) and, paradoxically, increases pain after repeated exposure to various opioids at doses that normally elicit analgesia. However, the molecular underpinnings of OIH are not well studied. Thus, further research into therapeutic modalities for the treatment of OIH during the perioperative period is warranted.

## Voltage-gated calcium channels and nociception

Several subtypes of voltage-gated calcium channels are expressed on neurons along the pain pathway and are crucial in action potentials and in controlling cellular excitability and synaptic transmission. On the basis of the membrane potential at which they become activated, these channels are subdivided into high-voltage-activated currents, and low-voltage-activated — or transient — calcium currents (3). These channels are products of different genes, which give rise to α1 subunits that form the pores of the voltage-gated calcium channels, named the Ca_V_1 family, which encodes L-type; Ca_V_2.1, encoding P/Q-type; Ca_V_2.2, encoding N-type; and Ca_V_2.3, encoding R-type. It is well established that N-type channel blockers have a major function in presynaptic inhibition in the dorsal horn of the spinal cord, and clinical studies are under way to establish their utility in treatments of patients with intractable pain. The cloning of T-type calcium channel (T-channel) pore-forming α1 subunits revealed the existence of at least three isoforms, called G (Ca_V_3.1), H (Ca_V_3.2), and I (Ca_V_3.3), and which are encoded by different genes, *cacna1g*, *cacna1h,* and *cacna1I*, respectively (4). It is well established that Ca_V_3.2 channels are essential for regulating excitability in nociceptors and promoting nociception in a variety of animal models (5–7), including rodents with plantar skin incision mimicking human surgical incisional pain (8, 9). In contrast, the T-channel Ca_V_3.1 isoform was implicated in trigeminal neuropathic pain (10).

## T-type channels regulate thalamocortical processing of sensory information

The thalamus is the major gateway for the flow of sensory information from the periphery to the cortex, and many drugs and pathological conditions associated with alterations in sensory processing may disrupt thalamocortical connectivity as an essential feature. Most thalamic neurons exhibit phasic behaviors, such as tonic and burst firing that represent different functional modes. During tonic firing, which predominates in awake states, there is a faithful transfer of sensory information to cortical neurons with characteristic low-amplitude, high-frequency EEG patterns. In contrast, during slow oscillations that occur with a T-channel-dependent, burst-firing pattern, sensory transfer becomes impaired and there is a gradual transition to sleep states. Hence, T-channels are crucial to the rhythmic oscillations between mutually interconnected cortical, inhibitory GABAergic interneurons and glutamatergic relay neurons in the ventrobasal thalamic nucleus. In most of the glutamatergic thalamic nuclei, the Ca_V_3.1 channel is the dominant subtype expressed on soma and dendrites. In contrast, in the thalamic reticular nucleus, which is composed from GABAergic neurons, the most abundantly expressed T-channel isoforms are Ca_V_3.2, which occur mostly on cell somas, and Ca_V_3.3, generally expressed on dendrites (11). One very important property of T-channels is their ability to open despite minimal depolarization; a low-voltage threshold sufficiently opens channels to allow activation of voltage-gated sodium channels and consequently the firing of action potentials. Importantly, unlike most other ion channels, T-channels deinactivate during neuronal hyperpolarization, a process that corresponds with recovery from inactivation, allowing them to open after depolarization and trigger a low-threshold calcium spike crowned with a barrage of action potentials. It is well documented that during natural sleep or general anesthesia, inhibitory synaptic inputs hyperpolarize thalamic cells sufficiently to recover T-channels from inactivation and consequently allow them to generate characteristic burst-firing and network oscillations.

## Thalamic Ca_V_3.1 channels and remifentanil-induced hyperalgesia

In this issue of the *JCI*, Yan Jin and colleagues (12) used an array of in vivo and ex vivo techniques to provide compelling evidence that hyperexcitable thalamocortical networks drive remifentanil-induced hyperalgesia in a rodent model of postsurgical pain. They discovered that glutamatergic neurons in the thalamic ventral posterior nucleus showed an increase in burst firing in a mouse model of surgical hind paw incision and remifentanil-induced hyperalgesia. The elegant study employed anatomical tracing, chemogenetics, and optogenetics to identify projection neurons in the hindlimb sensory cortex that mediate hyperalgesia. Furthermore, the authors used a combination of targeted pharmacological inhibition and in vivo knock-down to specifically identify an important role for the upregulated Ca_V_3.1 isoform of T-channels in this process. Although these findings are provocative, a few questions remain unanswered. For example, the current understanding of thalamic gate theory holds that burst firing in glutamatergic neurons should suppress the flow of sensory information, including pain. Conversely, inhibition of thalamic Ca_V_3.1 channels may cause hyperalgesia. Experimentally, global deletion of Ca_V_3.1 channels and infusion of T-channel inhibitor into ventroposterolateral thalamic neurons induced hyperalgesia to visceral pain caused by injections of magnesium sulfate or acetic acid (13). This finding strongly suggests that Ca_V_3.1 channels may affect neuronal excitability and inhibit or support pain signals from the periphery, depending on the placing and connectivity of neurons in the thalamus and the nature of painful stimuli. Another surprising finding in Jin et al. (12) was the absence of OIH with another opioid, sufentanil, which contrasts with clinical reports that associate the drug with OIH (1, 2). However, the discrepancy could be due to distinct pharmacokinetic properties of these drugs.

## Conclusion and clinical implications

Accumulating evidence suggests that the administration of opioid drugs leads not only to pain relief, but may also lead to a paradoxical sensitization of nociceptors (1, 2). This phenomenon is referred to as OIH and presents a substantial medical problem. Jin et al. (12) shed light on the molecular mechanisms of OIH and identify a critical thalamocortical circuit and the Ca_V_3.1 channel as a contributor in this process. Hence, it is tempting to postulate that thalamic T-channels may be targets for treatment of OIH associated with remifentanil. However, it remains to be seen if the same animal model will validate OIH with other opioids frequently used in perioperative periods, such as morphine. Furthermore, selective pharmacological inhibitors of T-type channel isoforms are not yet available. Nonetheless, exploring thalamic T channels as targets for the treatment of OIH remains an important area for future investigations.

## Version 1 12/15/2022

Electronic publication
